# Oxysterols and Retinal Degeneration in a Rat Model of Smith-Lemli-Opitz Syndrome: Implications for an Improved Therapeutic Intervention

**DOI:** 10.3390/molecules23102720

**Published:** 2018-10-22

**Authors:** Steven J. Fliesler, Libin Xu

**Affiliations:** 1 Departments of Ophthalmology and Biochemistry and Neuroscience Program, Jacobs School of Medicine and Biomedical Sciences, University at Buffalo, The State University of New York, Buffalo, NY 14260, USA; 2 Research Service, VA Western NY Healthcare System, Buffalo, NY 14260, USA; 3 Department of Medicinal Chemistry, School of Pharmacy, University of Washington, Seattle, WA 98195, USA

**Keywords:** antioxidant, cholesterol, degeneration, oxysterol, retina, Smith-Lemli-Opitz syndrome

## Abstract

Smith-Lemli-Opitz syndrome (SLOS) is an autosomal recessive human disease caused by mutations in the gene encoding 7-dehydrocholesterol (7DHC) reductase (DHCR7), resulting in abnormal accumulation of 7DHC and reduced levels of cholesterol in bodily tissues and fluids. A rat model of the disease has been created by treating normal rats with the DHCR7 inhibitor, AY9944, which causes progressive, irreversible retinal degeneration. Herein, we review the features of this disease model and the evidence linking 7DHC-derived oxysterols to the pathobiology of the disease, with particular emphasis on the associated retinal degeneration. A recent study has shown that treating the rat model with cholesterol plus suitable antioxidants completely prevents the retinal degeneration. These findings are discussed with regard to their translational implications for developing an improved therapeutic intervention for SLOS over the current standard of care.

## 1. Introduction

Since the discovery of cholesterol (Chol) by François Poulletier de la Salle in 1796 and its subsequent naming as “cholesterine” by Michel Eugène Chevreul in 1816 [1], the structure, biosynthesis, and biological functions of sterols have been the subject of extensive investigations [2,3,4,5,6]. Similarly, the study of oxysterols—oxidative derivatives of sterols—has emerged and evolved as a distinct research area of interest in chemistry, biology, and medicine [7,8,9,10,11,12,13]. Here, we present an overview of the formation, presence, and possible pathophysiological role of oxysterols in the retina in an animal model of a human cholesterol deficiency syndrome (see below), and the implications of these findings with regard to development of a new therapeutic intervention for this disease, based upon blocking the formation of cytotoxic oxysterols.

## 2. The RSH/Smith-Lemli-Opitz Syndrome (SLOS)

**Figure 1:**
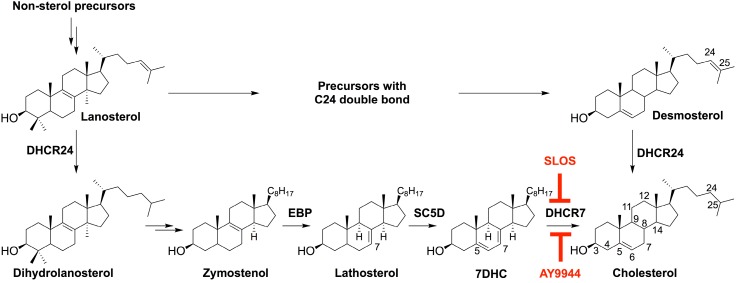
Post-squalene cholesterol biosynthetic pathway. Site of enzymatic inhibition by AY9944 is indicated as well as the locus of the defect in Smith-Lemli-Opitz syndrome (SLOS) at the level of DHCR7. Structures of cholesterol (Chol), 7-dehydrocholesterol (7DHC), and numbering for nomenclature are shown. EBP, 3β-hydroxysterol- ∆^8^,∆^7^-isomerase; DHCR24, 3β-hydroxysterol-∆^24^-reductase; SC5D, sterol-C5-desaturase; DHCR7, 3β-hydroxysterol-∆^7^-reductase.

RSH/Smith-Lemli-Opitz syndrome (SLOS) [14] is an autosomal recessive human genetic disease caused by a mutation-induced enzymatic defect in the last step in Chol synthesis, i.e., reduction of the ∆^7^-double bond of 7-dehydrocholesterol (7DHC) to form Chol, catalyzed by the enzyme, DHCR7 (7-dehydrocholesterol reductase; 3β-hydroxysterol-∆^7^-reductase, EC1.3.1.21) (Figure 1) [15,16,17,18,19]. This results in abnormally high steady-state levels of 7DHC and abnormally low levels of Chol in bodily tissues and fluids [15,20,21]; this biochemical phenotype is unique to SLOS and, hence, 7DHC is a signature biomarker for this disease. SLOS was the first characterized “multiple congenital anomalies (MCA)” syndrome, the first of several subsequently discovered inborn errors of Chol synthesis to be described over the past nearly six decades, and the most frequent Chol biosynthesis disorder with high carrier frequency (more than 1%) [16,19,22]. Features of this disease include multiple dysmorphologies involving craniofacial and musculoskeletal abnormalities (notably 2,3-toe syndactyly), brain malformation, impaired cognitive functions, autistic and other behavioral problems, developmental delay, and failure to thrive, among other defects [16,17,18,19,22]. Visual system dysfunction is also associated with SLOS [23,24,25].

## 3. AY9944 and the Development of a Rat Model of SLOS 

The causative link between excessive levels of blood-borne Chol, high-fat/high-Chol diets, and cardiovascular disease has been recognized since the 1950s (see ref. [3,4,5]). Hence, several pharmaceutical companies (e.g., Wyeth-Ayerst Laboratories, Merck, Eli Lily, Boehringer-Mannheim, etc.) have directed substantial efforts over the years toward developing Chol-lowering drugs, especially those that block the de novo synthesis of Chol—most notably, the statins, which block Chol synthesis at the level of HMG-CoA reductase, the main rate-limiting enzyme of the de novo pathway [26,27]. In addition, inhibitors that specifically target more distal enzymatic steps in Chol biosynthesis have been discovered, among them “AY9944” (*trans*-1,4-bis(2-chlorobenzylaminoethyl) cyclohexane dihydrochloride), which inhibits DHCR7—the same enzyme that is genetically abnormal in SLOS [28,29] (Figure 1). AY9944 was not successful as a cholesterol-lowering drug as it is teratogenic [30,31]. However, treating experimental animals, such as rats, with AY9944 has been employed successfully as a pharmacological approach for developing an animal model of SLOS [32]. We made modifications to the protocol originally developed by Kolf-Clauw et al. [32] to create an improved SLOS rat model that is viable for up to three postnatal months and exhibits profound elevation in 7DHC and reduction in Chol levels in the serum, liver, brain, and retina [33,34].

### 3.1. Retinal Degeneration in the AY9944-Induced SLOS Rat Model

**Figure 2:**
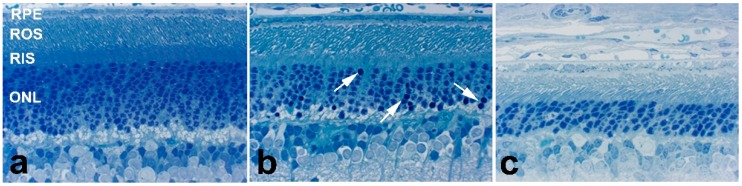
Histological degeneration of the retina observed in the AY9944-induced rat model of SLOS as a function of postnatal age at postnatal (**a**) one month, (**b**) two months, and (**c**) three months. Note progressive thinning of the outer nuclear layer (ONL), the appearance of pyknotic photoreceptor nuclei by two postnatal months (*arrows*, panel **b**), and the progressive shortening of rod outer segments (ROS). Epon embedment; toluidine blue stain.

The retina is rich in oxygen, cholesterol, light-absorbing retinoids, and polyunsaturated fatty acids (PUFAs), such as docosahexaenoic acid (DHA; 22:6), and is constantly exposed to light. Together, these factors make the retina susceptible to photo- or free radical-induced lipid oxidation and subsequent oxidative damage [35,36,37,38]. This is particularly relevant to this SLOS rat model because the accumulated cholesterol precursor, 7DHC, is highly prone to free radical oxidation [39] (next Section), which makes the retina even more susceptible to oxidative damage. Using the AY9944-induced SLOS rat model, we observed a progressive, irreversible, and profound retinal degeneration [34]. Remarkably, even though retina 7DHC/Chol mole ratios were >4:1 by one postnatal month, there were no appreciable structural abnormalities observed in the retina at that time point [33], although the retinal pigment epithelium (RPE) exhibited noticeable accumulation of phagosomes and membrane/lipid inclusions (Figure 2). However, by two postnatal months of treatment with AY9944, the retina exhibited marked pyknosis of the outer nuclear layer (ONL; the histological layer of the retina containing nuclei of rod and cone photoreceptor cells), degeneration of rod photoreceptor cells, and thinning of the neural retina. By three postnatal months, the severity of the retinal degeneration was more pronounced, including a loss of >25% of the photoreceptors (Figure 2). The degeneration appears to impact the photoreceptor layer almost exclusively. In fact, photoreceptor-specific cell death and dropout in this rat model has been confirmed independently, by TUNEL (terminal deoxynucleotidyl transferase dUTP nick end labeling) assay [40].

Consistent with this degenerative phenotype, the electrophysiological function of the retina was also progressively and markedly compromised in this rat model of SLOS, as determined by electroretinography (ERG) [34]. Although the amplitudes of rod- and cone-driven responses to light stimulation were robust and comparable to those of untreated control rats up to one postnatal month, response amplitudes were diminished, relative to controls, at two and three postnatal months, approximately proportional to the magnitude of retinal thinning and photoreceptor loss observed histologically. In addition, the timing of the photoresponses was much slower than normal.

### 3.2. Sterols and Oxysterols in the Retina in the AY9944-Induced Rat Model of SLOS

**Figure 3:**
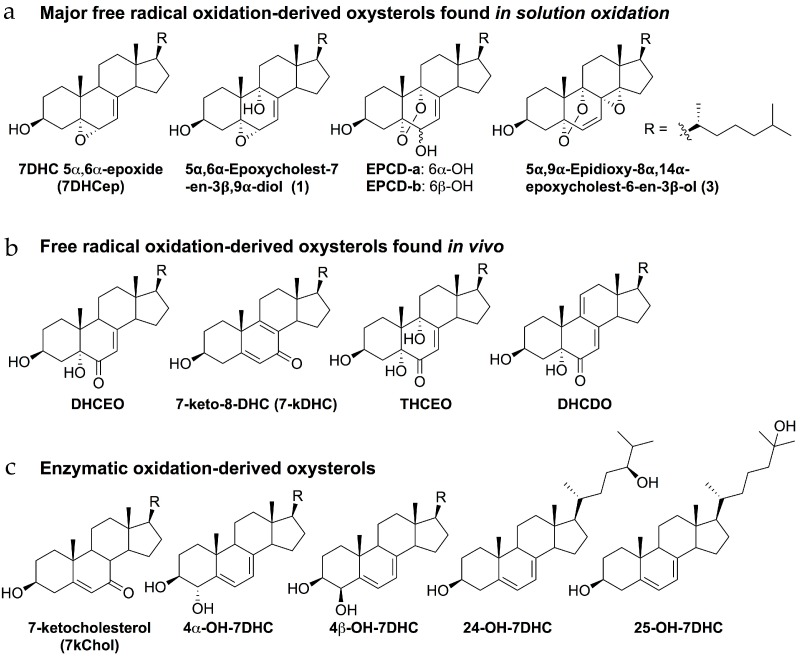
Chemical structure of some 7DHC-derived oxysterols: (**a**) Major oxysterols derived from free radical oxidation of 7DHC in organic solvent (benzene); (**b**) Oxysterols metabolites observed in biological systems that are derived from the primary oxysterols in solution; (**c**) Oxysterols derived from enzymatic oxidation of 7DHC by cytochrome P450 (CYP).

As mentioned above, the 7DHC/Chol mole ratio of retinas in this rat model become markedly elevated, relative to controls, with the ratio being about 4:1 at one postnatal month and increasing to >5:1 by three postnatal months [34]. Despite the apparent correlation between an increasing 7DHC/Chol mole ratio and severity of the observed retinal degeneration, however, there is no biological evidence to suggest that 7DHC itself is cytotoxic. In fact, 7DHC is almost identical to Chol in its physical properties: Its molecular mass, number of carbons, geometric shape, and molar volume are similar to those of Chol. Studies with artificial membrane bilayers, varying the sterol/phospholipid mole ratio as well as the relative proportions of 7DHC and Chol, have shown that the packing of 7DHC in membrane bilayers is nearly the same as that of Chol [41,42]. Furthermore, the ability of 7DHC to form “lipid rafts”—highly-ordered membrane microdomains containing high concentrations of sterols and sphingolipids, compared to the dominant bulk phase lipid composition—is comparable to, if not better than, Chol [43,44]. That said, there is a critical chemical difference between 7DHC and Chol: 7DHC has an additional double bond, which is also conjugated. That structural feature makes 7DHC highly susceptible to oxidation; in fact, it is the most highly reactive lipid molecule toward free radical oxidation [45,46,47]. In-solution oxidation of 7DHC leads to over a dozen oxysterols [45,46] (Figure 3a), many of which are further metabolized in biological systems, leading to metabolically more stable oxysterols [48,49] (Figure 3b). For example, 7DHC 5α,6α-epoxide (7DHCep) can be readily metabolized into 3β,5α-dihydroxycholest-7-en-6-one (DHCEO) while compounds, 5α,6α-epoxycholest-7-en-3β,9α- diol (1) and 5,9-endoperoxy-cholest-7-en-3β,6α(β)-diol (EPCD-a or -b), can serve as precursors to 3β,5α,9α-trihydroxycholest-7-en-6-one (THCEO) and 3β,5α-dihydroxycholesta-7,9(11)-dien-6-one (DHCDO) [49]. On the other hand, 7DHC is also prone to oxidation by cytochrome P450 (CYP) as a number of such metabolites have been identified in vitro and in vivo [49,50,51,52] (Figure 3c). In particular, 7-DHC can be directly converted to 7-ketocholesterol (7-kChol) by CYP 7A1, which is a novel mechanism of formation for this known cytotoxic oxysterol that is normally derived from Chol [50]. Indeed, both free radical and enzymatic oxidation-derived oxysterols, including DHCEO, 7-ketocholesterol (7-kChol), 4α-hydroxy-7DHC (4α-OH-7DHC), 4β-OH-7DHC, and 24-OH-7DHC, have been identified in retinas from AY9944-treated rats [52] (Figure 3).

Several of these 7DHC-derived oxysterols have been shown to be highly toxic to cells in culture, e.g., EPCD-a, DHCEO, and 7-kChol, while others exhibit little or no cytotoxicity [53]. Importantly, cell culture studies have shown that several of these 7DHC-specific oxysterols are highly toxic to retina-derived cells, and that transformed photoreceptor-derived 661W cells are preferentially more sensitive to such oxysterol-induced cytotoxicity compared to other types of retina-derived cells (e.g., RPE and glial cells) [54]. This latter point is consistent with the finding that the retinal degeneration in the AY9944-induced SLOS rat model appears to be photoreceptor-specific (see above). The molecular basis for this relative selectivity of oxysterol toxicity remains to be elucidated. Perhaps it is due to the fact that retinal photoreceptor cells are the most highly differentiated cells in the entire body, and maintain this rather unique status at the expense of certain protective mechanisms found in other cell types. Regardless, our findings are consistent with those obtained by other investigators, which have demonstrated that different cell types and tissues exhibit different degrees of sensitivity to the cytotoxic effects of oxysterols [53,55,56,57,58,59]. Furthermore, intravitreal injection of a small amount of 7kChol to rats led to massive retinal degeneration within one week [52], which is also consistent with the phenotype observed in AY9944-treated rats. Lipid hydroperoxide levels are also markedly elevated in the retinas of AY9944-treated rats, compared to untreated controls, under normal ambient lighting conditions [60], and exposure of those animals to intense constant light dramatically exacerbates both the increased levels of lipid hydroperoxides and the severity of the retinal degeneration [61]. Conversely, systemic pretreatment of the SLOS rat model with an antioxidant prior to exposure to intense constant light offers significant protection against the light-induced retinal degeneration, with concomitant reduction in the levels of lipid hydroperoxides [61].

**Figure 4:**
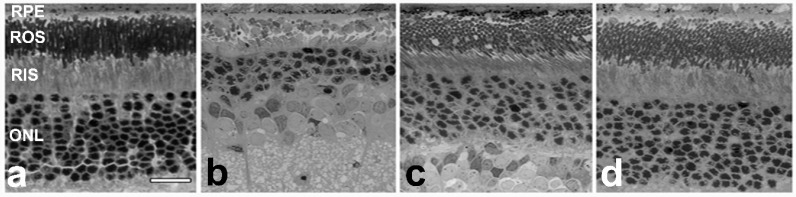
Retinal histology of normal control rat (**a**) and AY9944-treated (SLOS model) rats (**b,c,d**) as a function of dietary treatment (at postnatal three months of age). (**a**) Normal rat raised on standard (Chol-free) diet; (**b**) SLOS model rat raised on Chol-free diet; (**c**) SLOS model rat raised on 2%, by wt., Chol-enriched diet; (**d**) SLOS model rat raised on Chol-enriched diet supplemented with vitamins E and C and sodium selenite. Scale bar (*panel* **a**, for all panels), 20 μm. (*Adapted from Figure 1 in* [64] *with permission.*).

Taken together, these facts suggest that oxysterols (specifically those derived from 7DHC) may be causative in the retinal degeneration observed in the AY9944-induced SLOS rat model. If true, then blocking the formation of such oxysterols, e.g., with suitable antioxidants, should minimize or prevent the retinal degeneration from occurring [62,63]. Experiments to test that hypothesis have been performed and the results have been reported recently [64]. AY9944-treated adult rats were randomized into three groups: Group A rats were fed a Chol-free diet; Group B rats were fed a diet enriched in Chol (2 wt.%); Group C rats were fed a diet enriched in Chol (as for Group B) plus a mixture of water-soluble (vitamin C) and lipid-soluble (vitamin E) antioxidants and sodium selenite. A fourth group of rats, fed normal rat chow, served as untreated controls. At three posnatal months of age, electroretinograms were measured and then the rats were euthanized and their eyes were subjected to histological analysis. In good agreement with previous studies [34], Group A rats exhibited marked retinal degeneration and profoundly reduced ERG amplitudes, compared to untreated controls; feeding a high-cholesterol diet offered significant, but not complete, protection of retinas from degeneration, while feeding rats a diet enriched in both Chol and antioxidants provided remarkable and complete protection from retinal degeneration (no statistically significant differences between metrics of Group C and controls). The histological data from this experiment are provided in Figure 4.

Consistent with these findings, and with the hypothesis that 7DHC-derived oxysterols might be causative in the retinal degeneration, the retinas of Group A rats exhibited markedly elevated oxysterol levels (compared to untreated controls), Group B rat retinas showed a statistically significant lowering of oxysterol levels (compared to Group A), and Group C rat retinas exhibited additional reduction in total oxysterol levels (*ca.* 36% compared to Group A). We note that the main 7-DHC-derived oxysterols observed in retinas of AY9944-treated rats were presumably enzymatically derived oxysterols, i.e., 7-kChol, 4α-OH-7DHC, and 4β-OH-7DHC, as many primary oxysterols derived from free radical oxidation of 7DHC are highly electrophilic and could exist in the forms of adducts with nucleophilic residues of proteins [65]. Relative quantification of the amounts of protein adducts with 7-DHC-derived oxysterols in the retinas of AY9944-treated rats has not been performed, but increased protein adduction with a common ω-6 polyunsaturated fatty acid-derived electrophile, 4-hydroxynonenal (HNE), has been reported recently [66]. Regardless, the complete rescue of the retinal degeneration by the combination of Chol and antioxidants suggest that damages caused by 7DHC-derived oxysterols, through either recepter interactions or direct adduction with proteins, are mostly prevented. Detailed assessment of such adduct formation with or without antioxidant treatment would provide further support to this conclusion.

**Figure 5:**
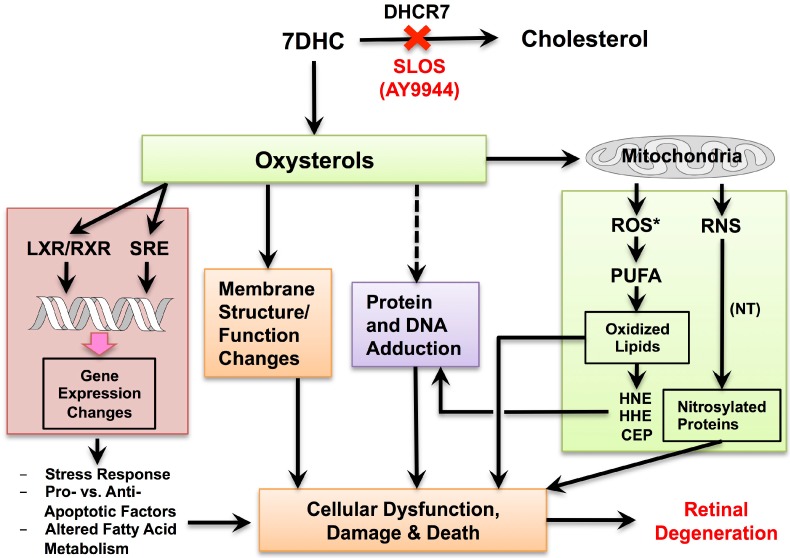
Schematic illustrating hypothetical mechanisms underlying retinal degeneration in the AY9944-induced rat model of SLOS and the therapeutic potential of antioxidants to prevent or minimize the degeneration. Abbreviations: LXR, liver X-receptor; RXR, retinoid X-receptor; SRE, sterol response elements; ROS*, reactive oxygen species; RNS*, reactive nitrogen species; HHE, 4-hydroxynonenal; HHE, 4-hydroxyhexenal; CEP, carboxyethylpyrrole; NT, nitrotyrosine; other abbreviations as described in text (*Adapted from Figure 29 in* [63] *with permission*.).

A summary schematic, depicting a hypothetical mechanism for the AY9944-induced retinal degeneration and the potential role of antioxidants as a therapeutic intervention to protect against this degeneration, is given in Figure 5. When DHCR7 activity is compromised, as occurs in SLOS and due to inhibition by AY9944, 7DHC accumulates and Chol levels become reduced, compared to untreated controls, in bodily tissues, including the retina. A portion of the 7DHC is then oxidized to form various oxysterols, some of which are highly toxic to cells, resulting in a myriad of sequellae—including protein damage by lipid electrophiles (including some oxysterols), gene expression changes, membrane structural and functional changes, formation of reactive oxygen species (ROS) and reactive nitrogen species (RNS), etc.—that collectively lead to photoreceptor dysfunction, degeneration, death, and dropout. However, blocking oxysterol formation with suitable antioxidants can minimize or prevent these sequellae and the ensuing retinal degeneration. The model assumes that this intervention would also entail Chol supplementation, in order both to provide the required endproduct of the pathway (Chol) as well as to suppress the formation of 7DHC (feedback inhibition of *de novo* synthesis at the level of HMG-CoA reductase).

## 4. Perspective and Future Directions

While the results obtained with combined antioxidant-Chol dietary supplementation using the AY9944-induced SLOS rat model are provocative, their application to therapeutic intervention in SLOS patients, especially neonates and children, should be considered with due caution. As previously pointed out [64], the potential toxicities of antioxidants in humans must be considered as well as appropriate scaling of dosages from the rodent model to humans. Considerable additional preclinical studies as well as clinical trials are needed before these results can be adapted for human use in treating SLOS patients, including determination of optimal types, amounts, frequencey, and route of administration of antioxidants, alone and in combination.

The relevance of the retinal degeneration observed in the AY9944-induced rat SLOS model to the human disease is somewhat speculative, due to several factors. First, there is only one publication extant that describes the retinal histopathology in a human SLOS patient [67]. The findings presented in that single case report were from post-mortem eyes obtained from a one-month old male child. While the retinas exhibited essentially normal stratification of the histological cell layers and differentiation of retinal cells types, including the rods and cones, the ocular tissue sections showed signs of substantial post-mortem artifacts. However, at this early postnatal stage, retinal degeneration similar to that observed in the AY9944 rat model would not be expected. [Note also that in the rat model, the retinas are histologically and electrophysiologically normal at one postnatal month (see discussion above).] There are only two published electrophysiological (ERG) studies of human SLOS patients: One reported slower than normal rod phototransduction activation and deactivation kinetics [24], while the second [25] reported normal cone photoreceptor function in this same patient cohort. However, these findings cannot be directly compared to the AY9944 rat SLOS model because all of those patients received standard-of-care (CHOL supplementation) therapy, whereas the rat model employs a CHOL-free dietary regimen. [Note that, unlike rats, human SLOS patients are not allowed (due to medical ethics considerations) to have this standard-of-care therapy witheld.] Also, no fundus photos or OCT (optical coherence tomography) retinal imaging were provided, so the histological status of the retina in these patients cannot be ascertained. Second, the human disease is genetic and the mutations are on-board from the inception of early embyogenesis; by contrast, the rat model is pharmacological and does not introduce the inhibitor of DHCR7 (AY9944) until the second gestational week (roughly equivalent in timing to onset of the second fetal trimester in humans). The AY9944 rat SLOS model is likely an approximation of what would occur in a severely affected SLOS patient in the absence of any kind of therapeutic supplementation. That model treated with a high-CHOL diet may more faithfully approximate the cellular and electrophysiological scenario in the more mildly or moderately affected human SLOS patients’ retinas.

In addition, the development of viable genetic mouse models of SLOS would be highly desirable to advance the field. Prior attempts to develop a global, homozygous *Dhcr7* knockout mouse have failed, due to early neonatal death (on postnatal day 0) [68,69]. Also, although a hypomorphic mutant *Dhcr7* knockin mouse line has been developed [70], it has been of limited value because its sterol profile progressively “self-corrects” over the first few postnatal months, for reasons that have yet to be elucidated with certainty. While treatment of normal animals with AY9944 or other DHCR7 inhibitors can mimic certain aspects of SLOS (notably the changes in tissue sterol profiles), it does not model the entire range of the SLOS phenotype and off-target pharmacological effects cannot be ruled out. Current studies are underway in our labs to develop targeted deletion of *Dhcr7* in specific retinal cell types in mice. A major obstacle to achieving this end has been the lack of a viable *Dhcr7^flx/flx^* mouse line. However, recently, that obstacle has been overcome and the targeted deletion of *Dhcr7* specifically in rod photoreceptor cells has been achieved (S.J. Fliesler, unpublished results). This breakthrough opens the way for targeted deletion of *Dhcr7*, not only in other retinal cell types (e.g., RPE cells, Müller glial cells, ganglion cells, etc.), but also in any cell type of interest throughout the body, in any tissue or organ (e.g., liver, brain, heart, kidney, etc.), using suitable available mouse lines that have targeted, promotor-driven expression of Cre recombinase in those cells, tissues, and organs of interest. We currently are embarking on studies to evaluate Chol homeostasis in the retina, with particular emphasis on retinal photoreceptor cells vs. the contributions made by other retinal cell types as well as blood-borne Chol to the total steady-state levels of Chol in the retina.
